# Shifting of global aphasia to Wernicke’s aphasia in a patient with intact motor function: a case report

**DOI:** 10.1186/s12883-021-02131-w

**Published:** 2021-03-11

**Authors:** Ya-Chi Chuang, Chuan-Ching Liu, I-Ching Yu, Yu-Lin Tsai, Shin-Tsu Chang

**Affiliations:** 1 Taichung Veterans General Hospital, Taichung, Taiwan; 2 Department of Physical Medicine and Rehabilitation, Taichung Veterans General Hospital, Taichung, Taiwan; 3 Department of Physical Medicine and Rehabilitation, Tri-Service General Hospital, Taipei, Taiwan; 4 School of Medicine, National Defense Medical Center, Taipei, Taiwan; 5 Department of Medicine, Chung Shan Medical University, Taichung, Taiwan

**Keywords:** Global aphasia without hemiparesis, Single photon emission computed tomography, Concise Chinese Aphasia Test

## Abstract

**Background:**

Global aphasia without hemiparesis (GAWH) is a rare stroke syndrome characterized by the dissociation of motor and language functions. Here, we present a case of GAWH with the patient later regaining speech fluency.

**Case presentation:**

A 73-year-old man was admitted to our emergency department immediately after an episode of syncope. On arrival, we noted his global aphasia but without any focal neurologic signs. Computed tomography (CT) perfusion scans showed a large hypodense region over his left perisylvian area. Under the impression of acute ischaemic stroke, he received recombinant tissue plasminogen activator (rtPA) injection and was treated as an inpatient. The patient was later discharged with GAWH status and received regular speech rehabilitation. After 14 months of rehabilitation, the patient gradually recovered his language expression ability. The degree of aphasia was evaluated with the Concise Chinese Aphasia Test (CCAT), and we obtained brain single photon emission computed tomography (SPECT) scans to assess cerebral blood flow.

**Conclusion:**

A patient with severe impairments of Broca’s and Wernicke’s areas was able to talk fluently despite being unintelligible. SPECT revealed relative high level of radioactivity uptake in the right frontal lobe, suggesting the deficits in speech fluency could have been compensated by the right hemisphere. Although this is a single case demonstration, the results may strengthen the role of the right hemisphere in GAWH patients and suggests additional study that examines the possible benefits of stimulating activity at right homologous regions for recovering language function after global aphasia.

## Background

Global aphasia without hemiparesis (GAWH) is a rare stroke syndrome. The incidence of GAWH was once mentioned as 0.32 % in ischaemic stroke patients by Pai et al. in 2011 [1]. It is manifested by global aphasia after large left perisylvian lesions [2]. The disorder is typically characterized by language impairment without contralateral hemiparesis. Earlier studies on GAWH investigated only the lesion site via brain MRI or CT in the acute phase [1, 3], and few studies discussed plastic changes in the brain during the chronic recovery phase. For adult Chinese individuals, the Concise Chinese Aphasia Test (CCAT) is a standardized and comprehensive test of language functions. In Taiwanese hospitals, this test is the most common and reliable test for assessing aphasia, including varying types of aphasia, and has benefits for quantifying communication disorders [4]. Here, we report a 73-year-old male Taiwanese patient with initial acute global aphasia without hemiparesis, and later recovery into Wernicke’s aphasia, with supporting evidence from the CCAT. Additionally, brain SPECT scans were obtained to assess cerebral perfusion during the chronic recovery phase.

## Case presentation

A 73-year-old right-handed Taiwanese man was admitted to our hospital after suffering syncope. On the day of admission, he had been well until 7:00 in the morning, when he was playing tennis. Specifically, he suddenly lost consciousness and fell to the ground. The patient promptly regained consciousness, but his verbal output was limited to saying the word “hao” (meaning “yes” in English). His friends took him to our emergency department at 8:39 am.

**Fig. 1:**
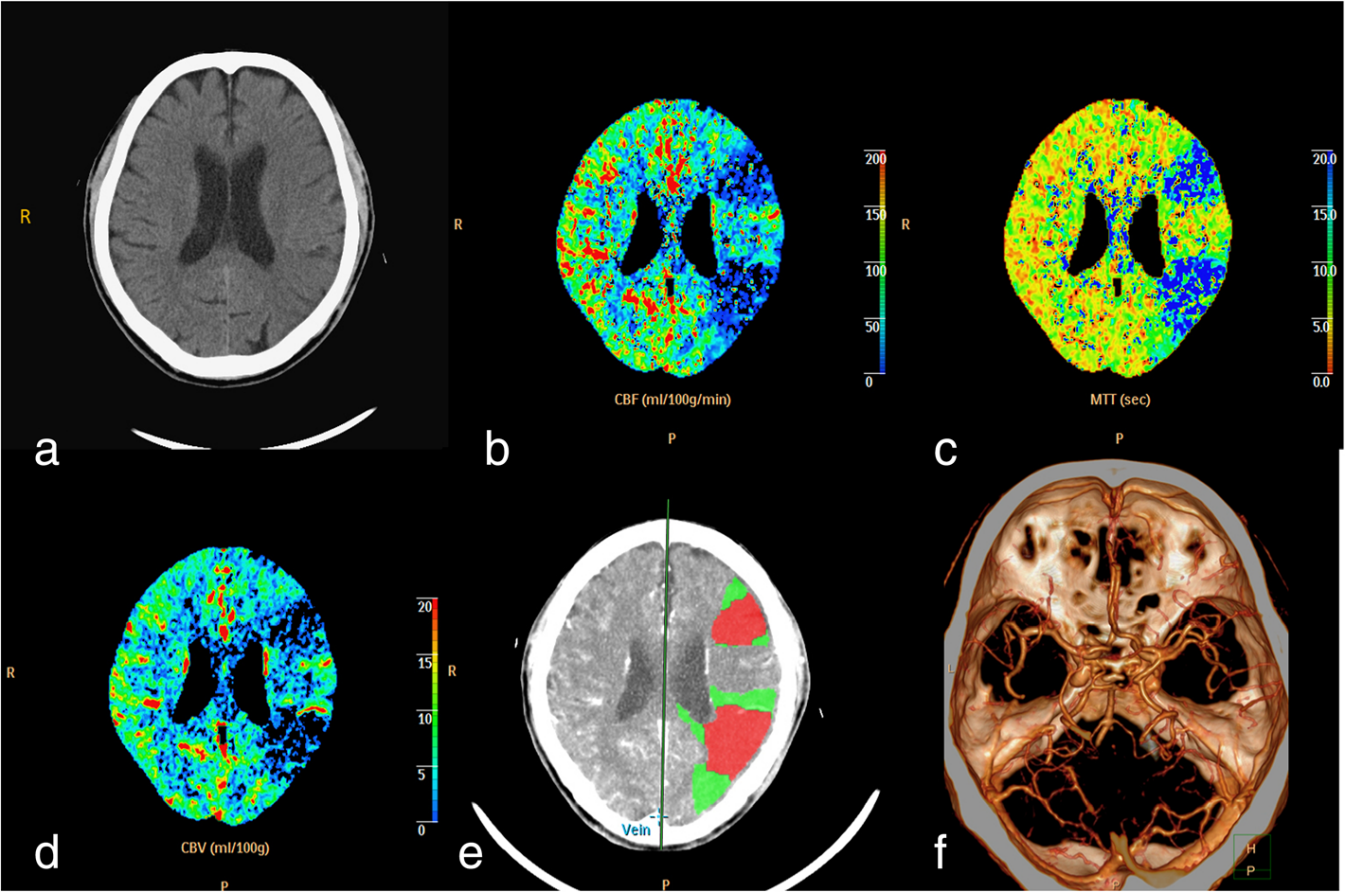
Brain CT and perfusion imaging-derived maps obtained in the emergency department. **a**: Pre contrast image showed no haemorrhage or obvious hypodense region. **b**: Cerebral blood flow (CBF). **c**: Mean transit time (MTT). **d**: Cerebral blood volume (CBV). **e**: CT perfusion (CTP). Core-penumbra colour map with estimated core and penumbra areas, highlighted in red and green, respectively. CTP shows a large deep core and penumbra regions over the left middle cerebral artery territory. **f**: CT angiography (CTA) showed no obvious vascular lesion on the main trunk of the intracranial arteries

On arrival, the patient was E2VaM4. On examination, he was alert. He had impairments in speech fluency, comprehension and repetition. His only verbal output was the sound “hao” or “hum”. Gait, extraocular motion, motor, and sensory functions were all normal. He showed no eye ptosis nor facial palsy. Blood parameters, including homocysteine levels, lipid profile, diabetes, oncology and endocrine measures, were unremarkable. Brain CT with contrast revealed no apparent intracranial haemorrhage or mass. However, CT perfusion indicated poor perfusion over the left middle cerebral territory (Fig. 1).

Tracing back his medical history, the patient was normostenic (body mass index was 18). He had hypertension for more than twenty years. He also suffered from hyperlipidaemia and coronary artery disease, and 7 months previously, he had received one drug-eluting stent. His daily medications included amlodipine 5 mg, aspirin 100 mg and rosuvastatin 5 mg. He played tennis twice a day and never smoked or drank alcohol. His highest education was senior high school and was fluent in Chinese, Taiwanese and Japanese. He used to work at an international trading company but had been retired for two years.

**Fig. 2:**
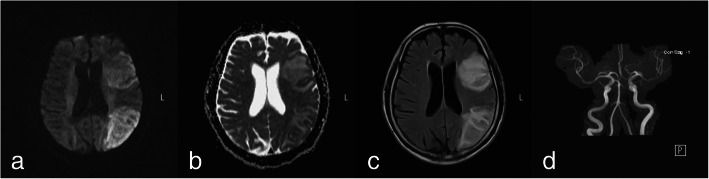
Brain magnetic resonance imaging on the fifth day after admission. **a**: Diffusion-weighted imaging (DWI) showed hyperintensity over the left frontal and temporoparietal regions. **b**: The apparent diffusion coefficient (ADC) image showed decreased ADC values in the same area. **c**: The T2-weighted fluid-attenuated inversion recovery (T2-FLAIR) image showed hyperintense regions in the same area. **d**: Magnetic resonance angiography (MRA) showed no obvious stenosis on the main trunk of the intracranial vessels. These findings combined with the clinical picture were consistent with acute ischaemic infarction

Under the impression of acute cerebral infarction with global aphasia, he then received a recombinant tissue plasminogen activator (rtPA) injection at 9:26 am and was switched to our intensive care unit for further management. Brain non-contrast magnetic resonance imaging (MRI; Fig. 2) was conducted on day 5, disclosing an acute infarction in the territory of the left middle cerebral artery (MCA), just in the perisylvian gyri, the classic areas of Wernicke and Broca. After 12 days of inpatient treatment, the patient was later discharged under stable conditions with global aphasia status and no focal neurologic signs. He was able to resume playing tennis on a daily basis soon after discharge.

**Fig. 3:**
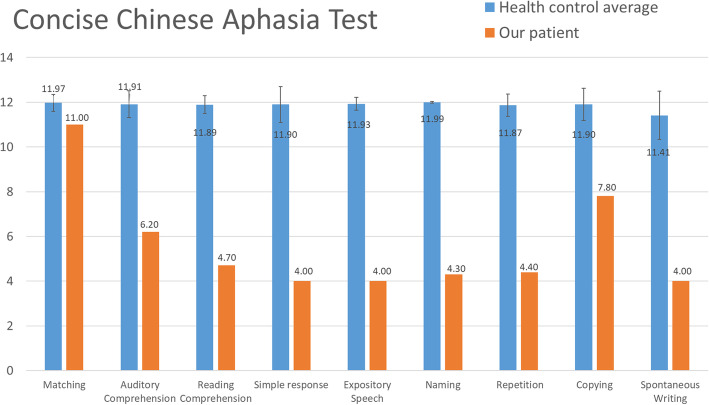
Profile of the Concise Chinese Aphasia Test (CCAT) 14 months after stroke onset. Blue bar: the average raw score obtained from 70 healthy control subjects based on Chung et al. [4]. Additionally, the error bar represents two standard deviations. Orange bar: our patient’s score on the CCAT

**Fig. 4:**
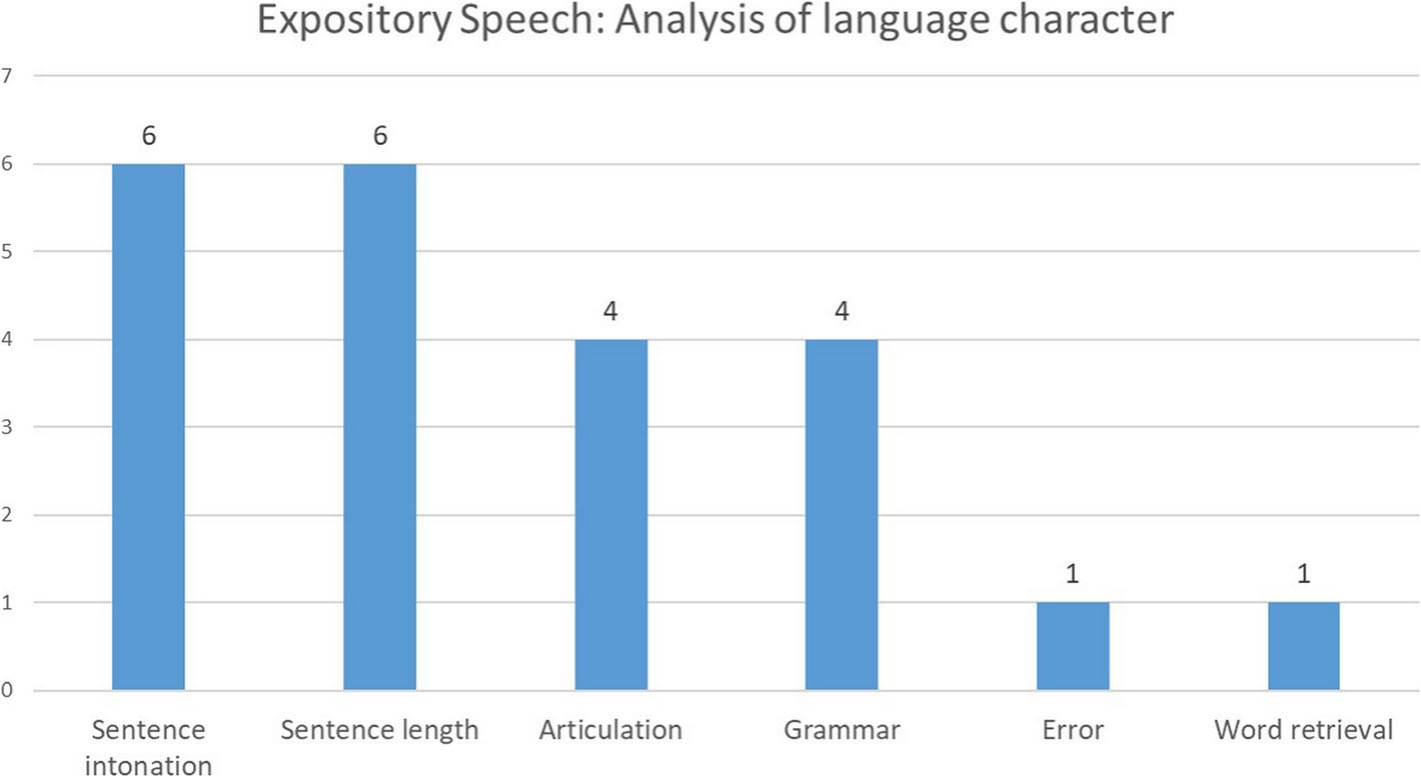
Profile of the analysis of expository speech on the Concise Chinese Aphasia Test. The average score in healthy people was 7. In this figure, we found that speech expression in our patient nearly reached healthy control levels in terms of sentence intonation and sentence length, suggesting that he had Wernicke’s aphasia

The patient then received speech rehabilitation, including verbal production, auditory comprehension training, augmentative communication training, phonetic placement and high-level cognitive function training at our hospital and another hospital. His language expression gradually recovered. The CCAT was then performed 14 months after the initial stroke. His average CCAT score was 5.6, indicating moderate to severe aphasia (Fig. 3). Detailed analyses of his spoken language characteristics showed subnormal performances for both sentence intonation and sentence length (Fig. 4), indicating he had fluent aphasia.

**Fig. 5:**
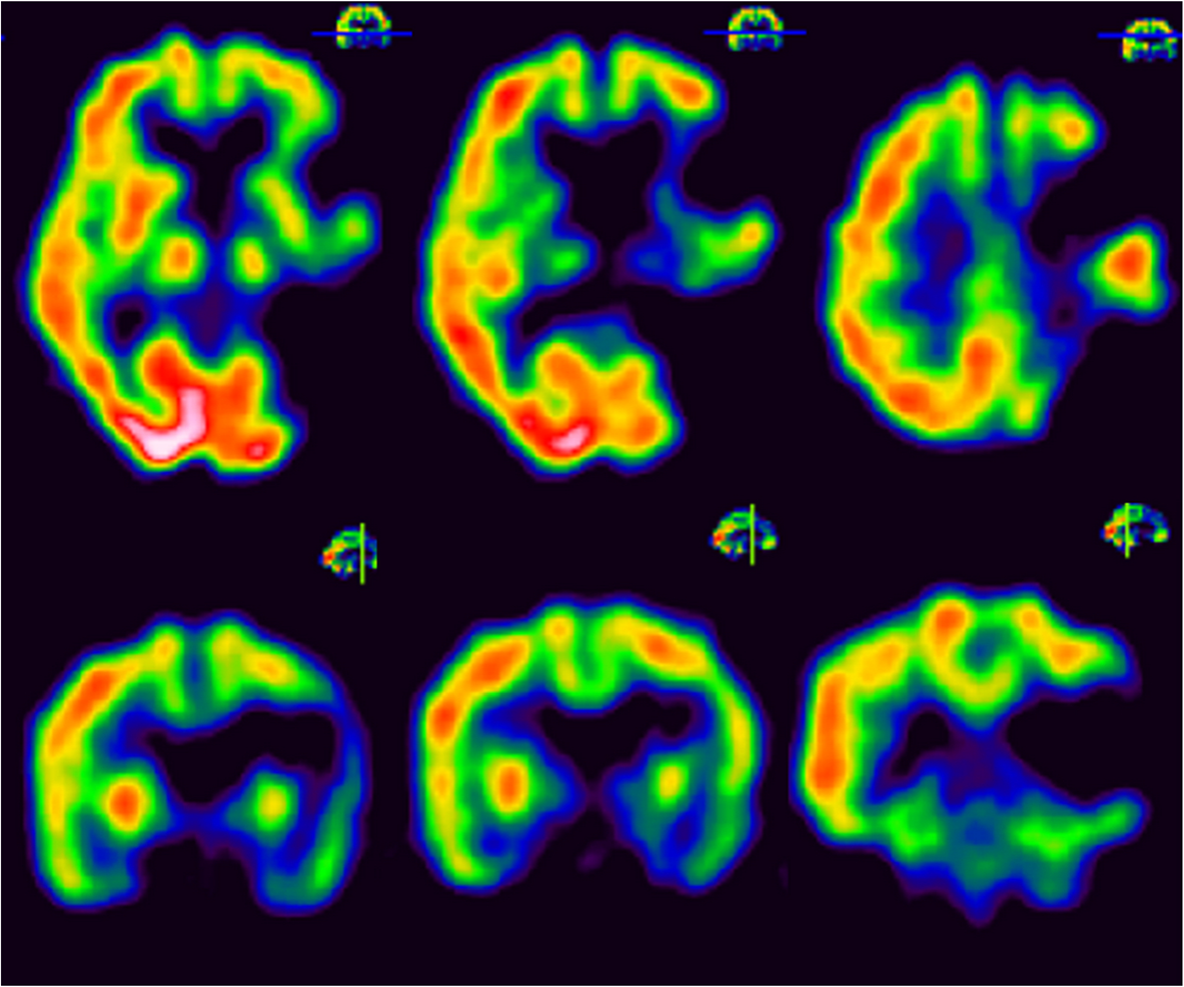
Brain single photon emission computed tomography (SPECT) image at 14 months after stroke onset. No radioactivity was seen in the left frontal and left parietotemporal regions. However, a relatively high level of radioactivity uptake was noted in the right thalamus, frontal and occipital regions

We subsequently checked is cerebral blood flow using regional brain single photon emission computed tomography (SPECT; Fig. 5). No radioactivity was found in the left frontal and left parietotemporal regions, and instead, a relatively high level of radioactivity uptake was noted over the right thalamus, frontal and occipital lobes.

## Discussion and conclusion

Here, we presented a case of Wernicke’s aphasia recovered from acute global aphasia without hemiparesis (GAWH), with supporting evidence from the CCAT. Both imaging tools, MRI in the acute phase and brain SPECT in the chronic recovery phase, showed a large infarction present over the left perisylvian regions involving both anterior and posterior language areas (Figs. 2 and 5). However, unlike the classic consensus that damage to both Broca’s and Wernicke’s areas results in global aphasia, our patient later showed only Wernicke’s aphasia. Although a previous study demonstrated that some patients present with an unexpected lesion-to-deficit correspondence, such as lesions in Broca’s area causing global aphasia [5] or lesions in the inferior and middle gyri causing Wernicke’s aphasia [6]. Our present case is the first to show severe impairments in the classic language network regions while preserving speech function.

It has been reported that there are better improvements in spoken language comprehension than speech production [7, 8]. However, in our case, we found the opposite results. One explanation is that GAWH is a unique stroke syndrome in which speech functions likely improve faster than sensory function, including language comprehension, through preserved motor structures in the brain. Mechanisms underlying recovery from aphasia could be coalesced into two principles by Stefaniak et al. (2020): (a) degeneracy and (b) variable neuro-displacement. In the theory of degeneracy, brain regions have the capacity to perform particular functions but will do so only when the original region underlying that function is damaged. With regard to variable neuro-displacement, a neural network utilizes its spare capacity and increases its performance in situations of increased difficulty [9]. Regarding the various mechanisms of recovery in different types of aphasia, Thomas et al. [10] suggested that during recovery in Broca’s aphasia, the initial right-hemispheric preponderance changed to left frontal lateralization, while in Wernicke’s aphasia, a presumably permanent shift towards the right hemisphere occurred.

Our case showed relatively high level of activity over the right hemisphere, especially in the thalamus and frontal and occipital lobes, and no left frontal lateralization was seen (Fig. 5). The results observed in our patient could be explained by the variable neuro-displacement theory, which differs from how language expression was previously thought to recover [10].

In 1999, Hanlon et al. investigated recovery from GAWH and proposed three types of GAWH: (a) persistent GAWH—global aphasia without any improvements in language functions, (b) GAWH-TCM—recovery from global aphasia to transcortical motor aphasia, and (c) GAWH-Wernicke’s—recovery from global aphasia with a shift to Wernicke’s aphasia [2]. Our case fell into the GAWH-Wernicke’s category. Based on the recovery mechanisms mentioned above and in line with the proposal of Hanlon et al., we speculated that the pattern of recovery in our patient was due to relatively higher activity in the right hemisphere that preserved motor function during a large stroke involving the MCA territory. Thus, during the 14-month recovery period, there was a faster recovery of speech function.

Previous studies, in particular, Jodzio et al. [11] and Mariën et al. [12], have provided discussions of the relationships between the recovery phase of aphasia and SPECT findings. The former studied 50 stroke patients with global aphasia and found extensive damage throughout the perisylvian region of the left hemisphere on SPECT. The latter reported a bilingual boy with acquired aphasia confirmed by SPECT, whose original lesion was in the left frontal lobe and left caudate nucleus, and remission of the deficit was associated with changes in the same areas.

In this case report, we presented a patient with recovery from global aphasia to fluent aphasia that was assessed by CCAT in support of the theory that the right hemisphere engages in language functions when the left perisylvian regions are severely impaired. Our findings provide insights into the role of the right hemisphere in GAWH patients. Further studies are needed to explore such patients and the plastic changes that occur in the right hemisphere that are homologous to the language regions following global aphasia. In the future, perhaps some interventions, such as repetitive transcranial magnetic stimulation, could be applied to these regions in the right (or left) hemisphere in this group of patients, thereby providing some benefits. Additionally, we should keep in mind that whenever we encounter GAWH patients, we should focus more on comprehension speech therapy for patients due to their delayed or poor recovery. Furthermore, brain SPECT is a good tool to evaluate the effectiveness of the treatment and is recommended for use by clinical doctors.

## Data Availability

The datasets used and/or analysed during the current study are available from the corresponding author on reasonable request.

## Abbreviations

GAWH: Global aphasia without hemiparesis
CT: Computed tomography
CCAT: Concise Chinese Aphasia Test
SPECT: Single photon emission computed tomography
MRI: Magnetic resonance imaging
MCA: Middle cerebral artery
MTT: Mean transit time
CTP: CT perfusion
